# Phylogeny and biogeography of the scaleless scale worm *Pisione* (Sigalionidae, Annelida)

**DOI:** 10.1002/ece3.2853

**Published:** 2017-03-22

**Authors:** Brett C. Gonzalez, Haidi Cecilie B. Petersen, Maikon Di Domenico, Alejandro Martínez, Maickel Armenteros, Erik García‐Machado, Peter Rask Møller, Katrine Worsaae

**Affiliations:** ^1^ Marine Biological SectionDepartment of BiologyUniversity of CopenhagenCopenhagen ØDenmark; ^2^ Center for Marine StudiesFederal University of ParanáParanáBrazil; ^3^ Molecular Ecology Group – Italian National Research CouncilInstitute for Ecosystem Study CNR‐ISEVerbania PallanzaItaly; ^4^ Centro de Investigaciones MarinasUniversidad de La HabanaHabanaCuba; ^5^ Evolutionary Genomics SectionNatural History Museum of DenmarkCopenhagen ØDenmark

**Keywords:** Aphroditiformia, comparative methods, copulatory structures, interstitial, morphological evolution, scale worm

## Abstract

*Pisione* is a scaleless group of small scale worms inhabiting sandy bottoms in shallow marine waters. This group was once considered rare, but now 45 described species can be characterized, among others, by their paired, segmental copulatory organs (one to multiple external pairs), which display a complexity of various accessory structures. The evolutionary significance of these unique organs was suggested in the late 1960s, but has been heavily debated since the late 1990s and remains controversial. In the present paper, we study the internal relationships within *Pisione,* employing combined phylogenetic analyses of both molecular and morphological data from 16 terminals of *Pisione,* as well as two terminals of *Pisionidens,* and eight additional scale worms as outgroups. Our taxon sampling covers all geographical areas where the genus has been reported, as well as most of their morphological and copulatory variability, including representatives of the “africana,” “remota,” “crassa,” and “papuensis” groups, established previously by Yamanishi. We hereby provide a first insight into the relationships of the genus, testing previously proposed hypotheses on the evolutionary significance of male copulatory structures within *Pisione*, while attempting to understand patterns of distribution. The phylogenetic analyses using maximum likelihood and Bayesian methods consistently recovered two large clades spanning the East Atlantic (including the Mediterranean) and the Indo‐Pacific–West Atlantic, respectively. Character optimization on our trees revealed a high degree of homoplasy in both non‐reproductive and sexual characters of *Pisione,* with buccal acicula found to be the sole apomorphy among the morphological features assessed herein, with none defining the biogeographical subclades within. Overall, our comparative analyses highlight the high degree of morphological variation in this widely distributed genus, rejecting previous assertions of an increasing number and complexity of copulatory structures across the genus.

## 1 Introduction

Since the mid‐nineteenth century, the placement of the small and aberrant annelid genus *Pisione* Grube, 1857 has been one of the trials and tribulations. Species of *Pisione* are unpigmented annelids, only a few millimeters in length, and with well over 50 segments. They are commonly found in sandy bottoms of shallow marine waters (Rouse & Pleijel, 2001), with one exception in freshwater (San Martín, López, & Camacho, 1998). However, their general morphology resembles various annelid groups, which accounts for the numerous suggested systematic affinities. *Pisione* was until recently one of four genera placed within Pisionidae *nomen suppressum*. A close association of this group to Aphroditiformia had long been proposed (i.e., Åkesson, 1961; Pleijel & Dahlgren, 1998). It was not, however, until 2005 that molecular and combined molecular and morphological analyses concluded that they are highly derived sigalionids (Struck, Purschke, & Halanych, 2005; Wiklund, Nygren, Pleijel, & Sundberg, 2005). A recent systematic analysis finally synonymized “Pisionidae” with Sigalionidae (Norlinder, Nygren, Wiklund, & Pleijel, 2012), a family within Aphroditiformia that includes scale‐bearing annelids with compound chaetae.

There are 46 recognized species and subspecies of *Pisione,* with the greatest numbers being described from throughout the tropical Indo‐Pacific Oceans (Salcedo, Hernández‐Alcántara, & SolíS‐Weiss, 2015; Yamanishi, 1998). *Pisione,* however, is not only restricted to this region and has likewise been found in tropical Atlantic and Caribbean waters (e.g., Martín, López, & Núñez, 1999; San Martín et al., 1998), the North Atlantic (Martínez, Aguirrezabalaga, & Adarraga, 2008), and Mediterranean (Aguado & San Martín, 2004). Regardless of the locality, this genus is commonly referred to as an interstitial group (Struck et al., 2005), whereas they might be better characterized as infaunal. Given their well‐developed parapodia and chaetae, it is unlikely that all pisionids can move among the sand grains without greatly displacing them (Giere, 2009; Higgins & Thiel, 1988; Swedmark, 1964). Members of *Pisione* were once considered rare (Hartman, 1959), yet the sheer number of recent descriptions indicates that the number of species will likely continue to increase (Aguado & San Martín, 2004; Martínez et al., 2008; Rouse & Pleijel, 2001).

**Figure 1:**
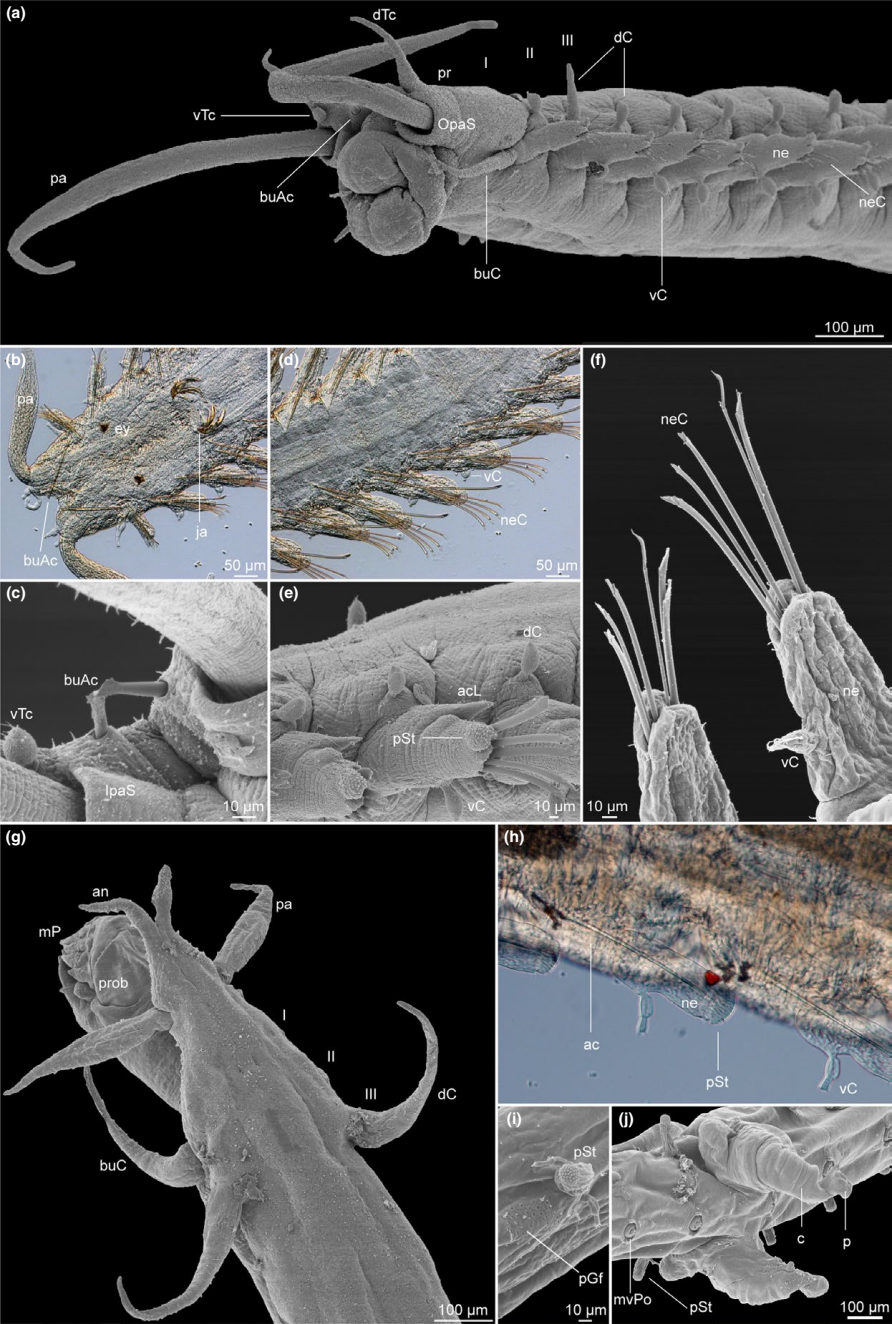
Morphological features of both *Pisione* Grube, 1857 (a–f) and *Pisionidens* Aiyar & Alikunhi, 1943 (g–j) used in the character coding and reconstructions. (a) *Pisione guanche* San Martín, López & Núñez, 1999 in lateral view with prostomial characters detailed. (b) *Pisione bulbifera* Yamanishi, 1998 with details of buccal aciculae, eyes, and jaws. (c) Buccal aciculae from *Pisione remota* (Southern, 1914). (d) Mid‐body segments of *P. bulbifera* including arrangement of neurochaetae. (e) Mid‐body neuropodia in *P. guanche,* showing the position of the acicular lobe and modified stylode with papillated/adhesive disks. (f) Example of notochaetae in *Pisione hartmannschroederae* Westheide, 1995. (g) Dorsal view of prostomial characters in *Pisionidens ixazaluohae* Petersen, Gonzalez, Martínez & Worsaae, 2016. (h) Detail of uniramous parapodia in *P. ixazaluohae* with internal aciculae visible and modified stylode with papillated/adhesive disks. (i) Detail of parapodial glandular field in *P. ixazaluohae*. (j) Detail of midventral pores, copulatory organ, and penis in *P. ixazaluohae*. Definitions of abbreviations: I–III, segment numbers; ac, aciculum; acL, acicular lobe; an, antenna; buAc, buccal aciculae; buC, buccal cirri; c, copulatory organ; dc, dorsal cirri; dTc, dorsal tentacular cirri; ey, eyes; ja, jaws; IpaS, inner palpal sheath; mP, mouth papillae; mvPo, midventral pores; ne, neuropodia; neC, neurochaetae; OpaS, outer palpal sheath; p, penis; pa, palps; pGf, parapodial glandular field; pr, prostomium; prob, proboscis; pSt, papillated stylode; vc, ventral cirri

Based on morphological comparisons, Yamanishi (1998) suggested that *Pisione* evolved from a *Pholoe*‐like ancestor. *Pisione* (Fig. 1) share with most sigalionids the presence of compound neurochaetae and a slender and elongated body, but lack scales (=elytra). The loss of scales is hypothesized as one of many adaptations to an interstitial lifestyle (Struck et al., 2005) and is a trait convergently shared by other interstitial scale worms including *Metaxypsamma* Wolf, 1986; as well as the macrofaunal *Palmyra* Savigny, 1818 (Watson Russell, 1989; Wiklund et al., 2005; Wolf, 1986). The interstitial lifestyle of *Pisione* also seems correlated with other changes, including copulation and internal fertilization. This reproductive strategy is commonplace to interstitial taxa (Giere, 2009), but unlike the external fertilization normally found in scale‐bearing macrofaunal annelids (Rouse & Pleijel, 2001). Reproductive adaptations are essential for interstitial annelids, especially with limited availability of reproductive products, body size, and space limitation within their environment (Jörger, Heß, Neusser, & Schrödl, 2009; Westheide, 1984; Yamanishi, 1998). Within *Pisione*, the males display elaborate paired copulatory organs, which may be present in a single segment, or up to 15 or more depending on the species. Yamanishi (1998) found these male copulatory structures to be essential in the classification of the group, while emphasizing that they could be informative for understanding the evolution and even biogeography of *Pisione*. However, due to immaturity or seasonality, penises may be lacking from the examined collected material, and Salcedo et al. (2015) have suggested that other non‐reproductive morphological characters may be systematically informative. While these structures would include characters like neurochaetae and buccal and neuroacicula, to date, no detailed study across taxa has compared the significance of these characters, nor of the copulatory organs.

**Figure 2:**
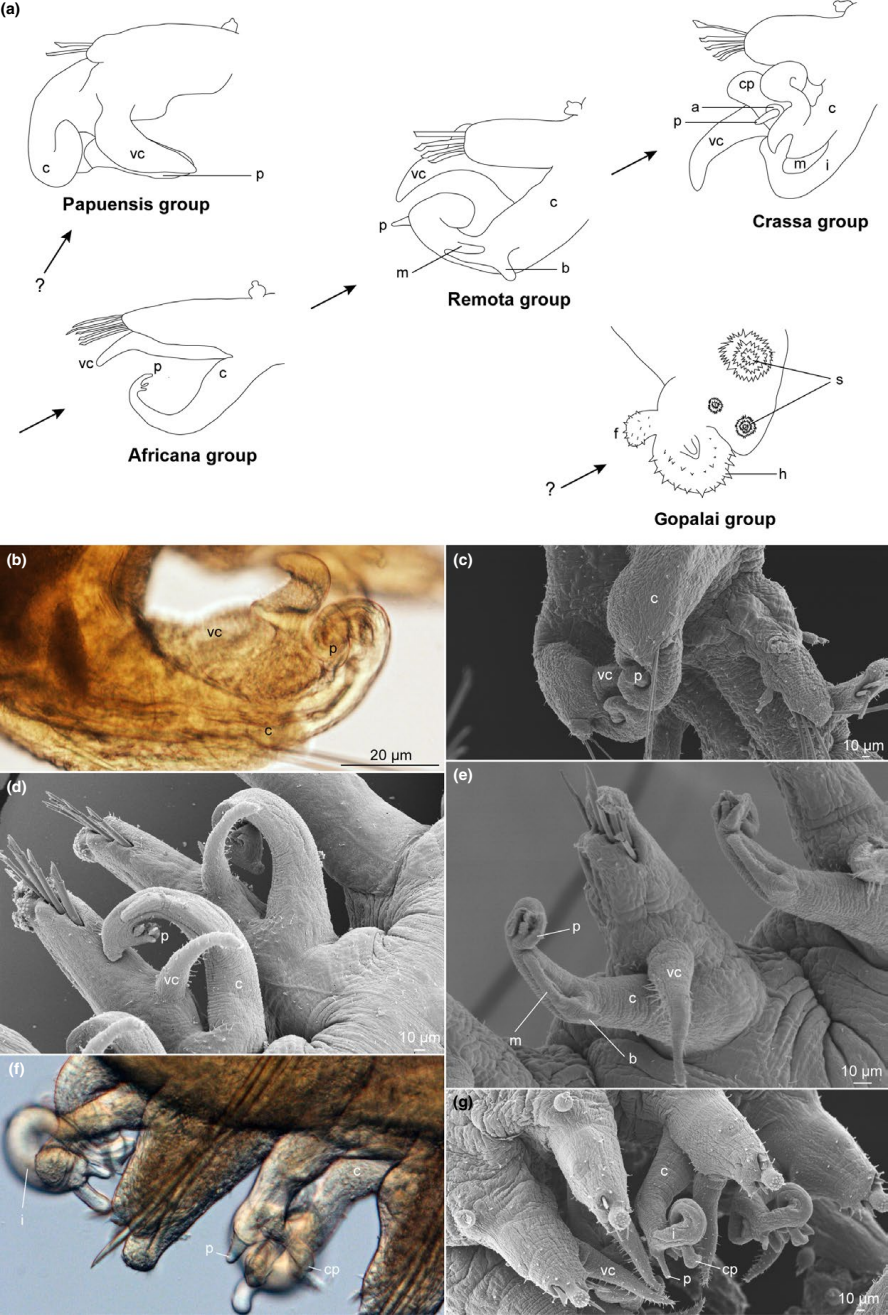
Male copulatory variability in *Pisione*. (a) Hypothesized evolutionary trend of male copulatory structures in *Pisione* (redrawn from Yamanishi, 1998). Arrows represent his suggested linear evolution from the “africana” group to the “crassa” group, while those with question marks (?) are outside the proposed evolutionary scheme. (b,c) LM and SEM images of *Pisione papuensis* Govaere & De Wilde, 1993 displaying male copulatory structures resembling that of the “papuensis” group. (d) SEM micrograph of *Pisione guanche* San Martín, López & Núñez, 1999 displaying male copulatory structures resembling the “africana” group. (e) SEM micrograph of male copulatory structures of *Pisione remota* (Southern, 1914), belonging to the “remota” group. (f,g) LM and SEM images of male copulatory structures of *Pisione* cf. *vestigalis* Yamanishi, 1998 of the “crassa” group. Definitions of abbreviations: a, sheath‐like arc; b, bidigitate process; c, copulatory organ; cp, cuticular plate; f, fan‐like appendage; h, hood; i, inferior stem; m, cuticular membrane; p, penis; s, spinous papillae; vc, ventral cirri

Yamanishi (1998) identified five groups based on a proposed evolutionary trend in male copulatory organization (Fig. 2). The simplest construct of copulatory structures was his “africana” group. According to Yamanishi, the “africana,” “remota,” and “crassa” groups evolved from an evolutionary progression in which accessory structures were added progressively to the copulatory organ, which consists of a thick and tapering copulatory structure adjacent to the ventral cirrus. Yamanishi (1998) further introduced two additional groups that did not fit into this proposed progression series. Still, his “papuensis” group does exhibit copulatory characters that resemble an intermediate between the “africana” and “remota” groups, whereas his “gopalai” group is characterized by the fusion of the copulatory organ stem and the parapodia, forming a bulging structure surrounded by a hood with spinous papillae. The ventral cirrus is greatly reduced in the “gopalai” group, but well developed in the “papuensis.” Regardless, these evolutionary hypotheses were strictly based on observations, and thus far, never investigated by phylogenetic methods.

We here investigate the evolutionary history of the genus *Pisione,* from both a phylogenetic and a biogeographical perspective. We implemented combined phylogenetic analyses to investigate the character evolution within the genus, while tracing the morphological character evolution on our tree topology. Two long‐standing questions were further addressed in our analytical comparisons. First, we compare the degree of homoplasy in both non‐reproductive and sexual characters in order to evaluate their diagnostic value for species identification. Second, we used comparative methods to investigate the detailed evolution of copulatory organs, testing the hypothesis of progressively increasing complexity in copulatory structures as proposed by Yamanishi (1998). And finally, we investigated the optimal distribution range for *Pisione,* testing for the presence of a latitudinal diversity gradient (LDG; Jablonski, Roy, & Valentine, 2006) and the preference of biogeographical hotspots (Bowen et al., 2013) based on all the records for the genus.

## 2 Materials and Methods

### 2.1 Specimen collection

Specimens for this study were collected on expeditions from 2007 to 2014 from Australia (2007), Belize (2010), Spain (2011, 2014, 2015), West Panama (2011), Brazil (2012), Italy (2013 and 2015), Indonesia (2013), Cuba (2014), Israel (2014), Sweden (2014), and México (2014) (See Table 1).

**Table 1:** All taxa included in the molecular and combined dataset analyses. When available, type/collection localities and accession numbers are provided

Family	Taxon	Coordinates	Collection locality	18S rDNA	28S rDNA	16S rDNA	COI	Copulatory group ^§^
Aph.	*Aphrodita aculeate*	–	Sweden	AY176281	JN852846	–	AY839578	–
	*Palmyra aurifera*	–	Pacific	AY83957	–	–	AY839583	–
Pho.	*Pholoe baltica*	–	Sweden	AY839573	JN852873	JN852912	AY839585	–
	*Pholoe pallida*	–	Norway	AY894302	JN852874	JN852913	AY894318	–
Pol.	*Harmothoe rarispina*	–	Greenland	**KY657611**	**KY657624**	**KY657641**	**KY657659**	–
Sig.	*Neoleanira tetragona*	–	Norway	AY839570	JN852872	JN852911	AY839582	–
	*Euthalenessa* cf. *digitata*	–	Casamicciola, Italy	**KY657612**	**KY657625**	**KY657642**	–	–
	*Pisionidens ixazaluohae*	–	Mexico	KX282503	KX282504	KX282502	KX282505	–
	*Pisionidens* sp.	–	Mexico	JN852842	JN852876	JN852915	JN852943	–
	*Pisione bulbifera*	29.544234/34.957981	Eilat, Israel	**KY657613**	**KY657626**	**KY657643**	**KY657660**	**Remota**
	*Pisione guanche*	29.155104/−13.427786	Lanzarote, Spain	**KY657614**	**KY657627**	**KY657644**	**KY657661**	**Africana**
	*Pisione hartmannschroederae*	22.602347/−78.666218	Cayo Guillermo, Cuba	**KY657615**	**–**	**KY657645**	**KY657662**	**Crassa**
	*Pisione hartmannschroederae*	19.982876/−75.866531	Miramar, Cuba	**–**	**KY657628**	**KY657646**	**–**	**Crassa**
	*Pisione hartmannschroederae*	16.80226/−88.081755	Carrie Bow Cay, Belize	**KY657616**	**KY657629**	**KY657647**	**–**	**Crassa**
	*Pisione hartmannschroederae*	−3.847415/−32.437092	Fernando de Noronha, Brazil	**KY657617**	**KY657630**	**KY657648**	**KY657663**	**Crassa**
	*Pisione hartmannschroederae*	19.968616/−76.408428	Chivirico, Cuba	**–**	**KY657631**	**KY657649**	**–**	**Crassa**
	*Pisione papuensis*	−21.917467/152.588242	Saumarez Reef, Australia	**KY657618**	**KY657632**	**KY657650**	**KY657664**	**Papuensis**
	*Pisione puzae*	40.577043/14.329735	Napoli, Italy	**KY657619**	**KY657633**	**KY657651**	**KY657665**	**Remota**
	*Pisione puzae*	37.029597/15.318359	Sicily, Italy	**–**	**KY657634**	**KY657652**	**KY657666**	**Remota**
	*Pisione* cf. *puzae*	43.488092/−8.322003	Galicia, Spain	**–**	**KY657635**	**KY657653**	**KY657667**	**Remota**
	*Pisione remota*	58.268694/11.410536	Lysekil, Sweden	**KY657620**	**KY657636**	**KY657654**	**KY657668**	**Remota**
	*Pisione* sp. A	−8.454662/144.439866	Raja Ampat, Indonesia	**KY657621**	**KY657637**	**KY657655**	**KY657669**	**–**
	*Pisione wolfi*	21.210457/−76.242444	Caletones, Cuba	**–**	**KY657638**	**KY657656**	**–**	**Crassa**
	*Pisione wolfi*	21.766186/−79.986889	Trinidad, Cuba	**KY657622**	**KY657639**	**KY657657**	**KY657670**	**Crassa**
	*Pisione* cf. *vestigalis*	−0.282247/−90.548478	Isla Bartolomé, Panama	**KY657623**	**KY657640**	**KY657658**	**KY657671**	**Crassa**
	*Sigalion spinosus*	–	California, USA	AY894304	DQ790062	–	AY894319	–

Taxa collected for this study along with their accessions numbers are listed in bold, while dashes (–) signify lack of gene coverage. Definitions of family abbreviations: Aph, Aphroditidae; Pho, Pholoidae; Pol, Polynoidae; Sig, Sigalionidae. Silcrow (*§*)—please refer to Yamanishi (1998) for detailed explanation of each male copulatory group designation.

All collections were carried out between the intertidal/swash zone and 30 m depth by snorkeling or diving. Specimens were extracted from fine sand to coral/volcanic rubble and gravel sediments. Animals were anesthetized in isotonic MgCl_2_ with seawater and extracted using the decantation method with a 63‐μm mesh (Pfannkuche & Thiel, 1988). Targeted specimens were sorted and identified to genus using a field microscope. Animals used for molecular analyses were preserved in 99% ethanol (EtOH) and stored at −20°C. Vouchers and specimens used for morphological character coding were fixed either in 3% glutaraldehyde or trialdehyde (in 0.1 mol/L cacodylate buffer with 5% sucrose), or in 2%–4% paraformaldehyde [PFA in PBS buffer; following protocols listed in (Kerbl, Bekkouche, Sterrer, & Worsaae, 2015)]. Original descriptions were used for taxonomic identification. Full species names and detailed collected localities are given in Table 1.

### 2.2 Morphological examinations

Morphological characters of all newly acquired material were examined using whole mounts prepared with glycerol on an Olympus IX70 inverted microscope mounted with an Olympus DP73 digital camera at the Marine Biological Section, University of Copenhagen, Denmark. Most morphological character coding was possible using light microscopy (LM) techniques (Table 2).

**Table 2:** Morphological characters used in the combined analyses. Character numbers, name, and states are provided as well as if they are treated as non‐reproductive versus sexual characters

No.	Character name	Type	Character states			
			0	1	2	3
1	Palp surface	Non‐reproductive	Smooth	Papillose	Ciliated	Rugose
2	Inner palpal sheath	Non‐reproductive	Absent	Present		
3	Outer palpal sheath	Non‐reproductive	Absent	Present		
4	Median antenna	Non‐reproductive	Absent	Present		
5	Lateral antenna	Non‐reproductive	Absent	Present		
6	Prostomial shape	Non‐reproductive	Absent	Present		
7	Prostomium	Non‐reproductive	Without lobes	Bilobed		
8	Tentacular cirri	Non‐reproductive	Absent	Present		
9	Eyes	Non‐reproductive	Absent	Present		
10	Segment 1	Non‐reproductive	Achaetous	Bearing chaetae		
11	Buccal acicula	Non‐reproductive	Absent	Present		
12	Notopodial sensory projection	Non‐reproductive	Absent	Present as dorsal cirri		
13	Position/distribution of the dorsal cirri	Non‐reproductive	Only on segment 3	On most non‐elytrigerous segments	On all segments	
14	Dorsal tubercles on non‐elytrigerous segments	Non‐reproductive	Absent	Present		
15	Parapodial form	Non‐reproductive	All uniramous	Biramous from segment 2		
16	Lateral glandular fields	Non‐reproductive	Absent	Present		
17	Notopodial stylodes	Non‐reproductive	Absent	Present		
18	Neuropodial stylodes	Non‐reproductive	Absent	Present		
19	Modified stylode with papillated/adhesive disks	Non‐reproductive	Absent	Present		
20	Parapodia modified for reproduction	Sexual^AG, RG, CG, PG, GP^	Absent	Present		
21	Proboscis/muscular pharynx	Non‐reproductive	Without chitinized structures	With chitinized structures		
22	Number of segments	Non‐reproductive	<50	>51		
23	Prechaetal lobes	Non‐reproductive	Undivided	Divided		
24	Notochaetae	Non‐reproductive	Absent	Present		
25	Simple neurochaetae	Non‐reproductive	Absent	Present		
26	Neurochaetal spines	Non‐reproductive	Absent	Present		
27	Unilateral fringed neurochaetae	Non‐reproductive	Absent	Present		
28	Compound falcigerous neurochaetae	Non‐reproductive	Absent	Present		
29	Compound spinigerous neurochaetae	Non‐reproductive	Absent	Present		
30	Elytra	Non‐reproductive	Absent	Present		
31	Midventral pores	Non‐reproductive	Absent	Present		
32	Protruding notoacicula	Non‐reproductive	Absent	Present		
33	Infra‐acicular simple chaetae	Non‐reproductive	Absent	Present		
34	Elongation of dorsal cirri on segment 3	Non‐reproductive	Absent	Present		
35	Prechaetal lobes divided	Non‐reproductive	Absent	Present		
36	Long‐bladed compound chaetae	Non‐reproductive	Absent	Present		
37	Fusion of copulatory organ and parapodial lobe	Sexual^PG^	Absent	Present		
38	Spiral structure of the copulatory organ	Sexual^RG, CG, PG^	Absent	Present		
39	Inferior stem of copulatory organ derived from bidigitate process	Sexual^AG, RG, CG^	Absent	Present		
40	Bidigitate process of copulatory organ homologous to inferior stem	Sexual^AG, RG, CG^	Absent	Present		
41	Sheath‐like arc of the copulatory organ	Sexual^AG, RG, CG^	Absent	Present		
42	Cuticular plate of the copulatory organ	Sexual^RG, CG^	Absent	Present		
43	Elongated ventral cirri of the copulatory segments	Sexual^RG, CG^	Absent	Present		
44	Spinous papillae present on copulatory structures	Sexual^PG^	Absent	Present		

Characters that may be associated with groups designated by Yamanishi (1998), and scored for species included in our dataset, are listed in superscript and include: AG, “africana” group; RG, “remota” group; CG, “crassa” group; PG, “papuensis” group; GG, “gopalai” group.

Specimens requiring detailed examination of copulatory segments, chaetal characters, and other gross anatomy were prepared for scanning electron microscopy (SEM). Specimens prepared for SEM were first transferred to cacodylate buffer, post‐fixed in 1% osmium tetroxide (in 0.1 mol/L cacodylate solution) for 1 h, and rinsed in distilled water. Specimens were dehydrated using a graded ethanol series (20%–100%) and transferred over three graded steps to 100% acetone for critical‐point‐drying. Critical‐point‐dried specimens were mounted on aluminum stubs and sputter‐coated with platinum/palladium using a high‐resolution fine coater (JFC‐2300HR) and examined using a JEOL JSM‐6335F field emission scanning electron microscope at the National History Museum of Denmark, University of Copenhagen.

### 2.3 Morphological data matrix

Forty‐four morphological characters (Fig. 1; Table 2) were used to construct a morphological data matrix of 16 ingroup taxa, including their sister taxa *Pisionidens* Aiyar & Alikunhi, 1943, as well as several additional scale worms based on both direct observations and a review of the literature (Salcedo et al., 2015; Yamanishi, 1998). Detailed character descriptions and their states are listed in Appendix 1. Characters were selected based on ongoing phylogenetic studies on the relationships between Aphroditiformia families and major clades (Gonzalez, Petersen, Martinez, & Worsaae, 2015), as well as previous reviews of the genera *Pisione* and *Pisionidens* (Petersen, Gonzalez, Martínez, & Worsaae, 2016; Salcedo et al., 2015; Yamanishi, 1998). The final matrix of scored characters for all taxa included in this study is listed in Table 3.

**Table 3:** Morphological data matrix of all 44 characters and taxa used in the combined analysis

	Character number	5					10					15					20					25					30					35					40					44			
Aph.	*Aphrodita aculeate*	1	0	0	1	0	1	0	1	1	1	0	1	2	1	1	0	0	0	0	0	0	0	0	1	1	1	0	0	0	1	0	0	0	–	–	–	–	–	–	–	–	–	–	–
	*Palmyra aurifera*	2	0	0	1	0	1	0	1	1	1	0	1	2	0	1	0	0	0	0	0	0	0	0	?	1	0	0	0	0	0	0	1	0	–	–	–	–	–	–	–	–	–	–	–
Pho.	*Pholoe baltica*	0	0	0	1	?	0	1	1	1	0	0	0	–	?	1	0	0	0	0	0	1	1	0	1	0	0	1	1	0	1	0	1	0	–	–	0	–	–	–	–	–	–	–	–
	*Pholoe pallida*	0	0	0	1	0	0	0	1	0	0	0	0	–	1	1	0	0	0	0	0	1	1	0	1	0	0	1	1	0	1	0	1	0	–	–	0	–	–	–	–	–	–	–	–
Pol.	*Harmothoe rarispina*	1	0	0	1	1	0	1	1	1	0	0	1	2	1	1	0	0	0	0	0	?	0	0	1	1	1	0	0	0	1	0	1	0	–	0	–	–	–	–	–	–	–	–	–
Sig.	*Neoleanira tetragona*	0	1	1	1	1	1	0	1	0	1	0	1	0	1	1	0	1	1	0	0	1	1	0	1	0	0	0	0	1	1	0	0	0	–	–	1	–	–	–	–	–	–	–	–
	*Euthalenessa* cf. *digitata*	0	1	1	1	1	0	0	1	1	1	0	1	0	1	1	0	1	1	0	0	1	1	0	1	0	0	0	1	0	1	0	0	0	–	–	1	–	–	–	–	–	–	–	–
	*Pisionidens ixazaluohae*	0	0	0	0	1	0	0	0	1	0	0	1	3	0	0	1	0	1	1	1	1	1	0	0	–	–	–	–	–	0	1	0	–	1	–	–	0	0	1	1	1	0	0	0
	*Pisionidens* sp.	0	0	0	0	1	0	0	0	1	0	0	1	3	0	0	1	0	1	1	1	1	1	0	0	?	–	–	–	–	0	1	0	–	1	–	–	?	?	?	?	?	?	?	?
	Pisione bulbifera*	3	1	1	0	0	0	1	1	1	0	1	1	3	0	1	0	0	1	1	1	1	1	1	0	1	1	1	1	1	0	0	0	0	0	1	1	0	0	0	0	0	0	1	0
	*Pisione guanche*	3	1	1	0	0	0	1	1	1	0	1	1	3	0	1	0	0	1	1	1	1	1	1	0	1	1	1	1	1	0	0	1	0	1	0	0	0	?	1	1	1	0	0	0
	*Pisione hartmannschroederae*	3	1	1	0	0	0	1	1	1	0	1	1	3	0	1	0	0	1	1	1	1	1	0	0	1	1	1	1	1	0	0	0	0	1	1	1	0	0	0	0	0	1	1	0
	*Pisione hartmannschroederae*	3	?	?	0	0	0	1	1	1	0	1	1	3	0	1	0	0	1	1	1	1	1	1	0	1	1	1	1	1	0	0	0	0	0	0	1	?	?	?	?	?	?	?	?
	Pisione hartmannschroederae*	3	1	1	0	0	0	1	1	1	0	1	1	3	0	1	0	0	1	1	1	1	1	0	0	1	1	1	1	1	0	0	0	0	1	1	1	0	0	0	0	0	1	1	0
	Pisione hartmannschroederae*	3	1	1	0	0	0	1	1	1	0	1	1	3	0	1	0	0	1	1	1	1	1	?	0	1	1	1	1	1	0	0	?	?	?	?	?	?	?	?	?	?	?	?	?
	Pisione hartmannschroederae*	3	1	1	0	0	0	1	1	1	0	1	1	3	0	1	0	0	1	1	1	1	1	?	0	1	1	1	1	1	0	0	?	?	?	?	?	?	?	?	?	?	?	?	?
	*Pisione papuensis*	3	?	?	0	0	0	1	1	1	0	1	1	3	0	1	0	0	1	1	1	1	1	0	0	1	1	1	1	1	0	0	0	0	1	1	1	2	1	0	0	0	0	0	1
	Pisione puzae*	3	1	1	0	0	0	1	1	1	0	1	1	3	0	1	0	0	1	1	1	1	1	1	0	1	1	1	1	1	0	0	0	1	0	0	0	0	1	0	1	?	?	1	0
	*Pisione puzae*	3	1	1	0	0	0	1	1	1	0	1	1	3	0	1	0	0	1	1	1	1	1	0	0	1	1	1	1	1	0	0	0	0	1	1	1	?	1	1	1	1	1	1	0
	Pisione puzae*	3	1	1	0	0	0	1	1	1	0	1	1	3	0	1	0	0	1	1	1	1	1	?	0	1	1	1	1	1	0	0	0	1	0	0	0	0	1	0	1	?	?	1	0
	*Pisione remota*	3	1	1	0	0	0	1	1	1	0	1	1	3	0	1	0	0	1	1	1	1	1	1	0	1	1	1	1	1	0	0	0	1	0	0	0	0	1	0	1	1	1	1	0
	*Pisione* sp. A	?	?	?	0	0	0	1	1	1	0	?	1	3	0	1	0	0	1	1	1	1	1	?	0	1	1	?	1	1	0	0	0	1	0	0	0	0	1	1	0	1	1	1	0
	*Pisione wolfi*	3	1	0	0	0	0	1	1	1	0	1	1	3	0	1	0	0	1	1	1	1	1	0	0	1	1	1	1	1	0	0	0	0	0	1	1	0	1	1	1	0	0	1	0
	Pisione wolfi*	3	1	1	0	0	0	1	1	1	0	1	1	3	0	1	0	0	1	1	1	1	1	?	0	1	1	1	1	1	0	0	0	0	0	1	1	0	1	1	1	0	0	1	0
	*Pisione* cf. vestigalis*	3	1	1	0	0	0	1	1	1	0	1	1	3	0	1	0	0	1	1	1	1	1	1	0	1	1	1	1	1	0	0	0	1	0	1	0	0	1	1	0	1	1	1	0
	*Sigalion spinosus*	0	0	0	?	?	0	0	1	1	?	0	0	–	?	1	0	0	0	0	0	1	1	0	1	?	0	0	1	0	1	0	?	0	–	–	1	–	–	–	–	–	–	–	–

Species marked with an asterisk (*) have characters 32–44 scored only from original descriptions. Unknowns are marked by a question mark (?), and inapplicable states are marked by a dash (–). Definitions of family abbreviations: Aph, Aphroditidae; Pho, Pholoidae; Pol, Polynoidae; Sig, Sigalionidae.

The 44 morphological characters included 39 binary and five multistate characters treated as independent and unordered. Following the principles of “c‐coding,” linked characters were coded and treated hierarchically (Pleijel, 1995). Each character was coded as “absence/presence” with linked traits subsequently coded as a multistate character or as “inapplicable” (–). Inapplicable characters (–) and missing data (?) were differently coded in order to facilitate the evaluation of our character coding even though the analyses treat them equally.

### 2.4 Taxon selection

The positions of *Pisione* and *Pisionidens* were recently solved in an extensive study that included representatives from all families of Aphroditiformia. Based on this analysis, we chose outgroup taxa from closely and more distantly related lineages, including within sigalionids, *Euthalenessa* cf. *digitata*;* Neoleanira tetragona* (Örsted, 1845); *Pholoe baltica* Örsted, 1843; *Pholoe pallida* Chambers, 1985; and *Sigalion spinosus* Hartman, 1939; polynoid *Harmothoe rarispina* Sars, 1861, and two aphroditids, *Aphrodita aculeata* Linnaeus, 1758 and *Palmyra aurifera* Savigny in Lamarck, 1818.

Our *Pisione* samples included representatives from all copulatory morphotypes designated by Yamanishi (1998) except for the “gopalai” group. Geographically, our samples span all but the polar oceans (where the genus has never been recorded), including representatives from the Caribbean, Mediterranean, and Red Seas. All previously published sequences for *Pisione* were included in the phylogenetic analyses, even if they potentially represent the same morphological species (e.g., *Pisione wolfi* San Martín, López & Núñez, 1999) (Table 1). The only exception to this was *Pisione guanche* San Martín, López & Núñez, 1999 from Tenerife and Lanzarote, which was removed a priori based on identical morphology and genetic sequences across all molecular markers. Specimens from the same geographical region for which morphological observations were not possible or where the morphology was highly similar were furthermore investigated for their taxonomical distinctiveness using species delineation analyses of their DNA sequences (see below).

### 2.5 Molecular techniques

Whole genomic DNA was extracted from 5 to 15 segments using the Qiagen DNeasy Blood & Tissue Kit (Qiagen Inc., Valencia, CA, USA) following the manufacturer's protocol. DNA elution was in 160 μl of buffer, and elution step was repeated with original buffer to optimize DNA yield.

Amplification reaction mixtures totaled 25 μl and employed either PuReTaq Ready‐To‐Go PCR beads (GE Healthcare Life Sciences, Buckinghamshire, UK), or GoTaq Green master mix (Promega Corporation, Madison, WI, USA). Reaction mixtures contained 1× Ready‐To‐Go beads, 1 μl of each primer (10 μmol/L concentration ea.), 21 μl Milli‐Q water, and 2 μl DNA template. Mixtures using GoTaqGreen contained 12.5 μl GoTaq Green, 1 μl of each primer (10 μmol/L concentration ea.), 9.5 μl Milli‐Q water, and 2 μl DNA template.

Approximately 1,850 base pairs (bp) of the small ribosomal RNA subunit (18S rDNA) were amplified using three overlapping fragments using the following paired primer sets (Giribet, Carranza, Baguñà, Riutort, & Ribera, 1996): (1) 18S1f/18S5R, (2) 18S3F/18Sbi, and (3) 18Sa2.0/18S9R. Fragments of the large subunit ribosomal RNA 28S rDNA D1‐D3 fragment (*ca*. 1,000 bp) were amplified using 28SG758 (Brown, Rouse, Hutchings, & Colgan, 1999) and 28SD3 (Vonnemann, Schrödl, Klussmann‐Kolb, & Wägele, 2005). The 16S ribosomal RNA (16S rDNA; *ca*. 500 bp) was amplified using the primer set 16SarL/16SbrH (Palumbi, 1996), and *ca*. 650 bp of the mitochondrial protein‐coding gene cytochrome c oxidase subunit I (COI) was amplified using the primer set dgLCO1490/dgHCO2198 (Meyer, 2003). All reactions were heated in a Bio‐Rad S1000 Thermal Cycler following primer specific temperature profiles.

Polymerase chain reactions (PCR) gene fragments were visualized on a 1% agarose gel stained with GelRed^™^ (Hayward, CA, USA). PCR products were purified using the E.Z.N.A. Cycle‐Pure Kit (Norcross, GA, USA) using 40 μl of elution buffer. Purified products were sequenced on an ABI 3730XL DNA Analyser (Applied Biosystems, Foster City, CA, USA) by Macrogen Europe (Amsterdam, the Netherlands).

All chromatogram readings and contig assembly were carried out in Sequencher 4.10.1 (GeneCodes Corporation, Ann Arbor, MI, USA). Contigs were checked for contamination using NCBI BLAST, and final sequences were visualized and trimmed pre‐ and post‐alignment using BioEdit (Hall, 1999).

Newly generated sequences were deposited in GenBank with the accession numbers KY657611‐KY657671 (Table 1).

### 2.6 Alignment and dataset assembly

All genes were aligned individually using the MAFFT online platform (Katoh & Standley, 2013), using the algorithm E‐INS‐I iterative refinement method (Katoh, Kuma, Toh, & Miyata, 2005; Kuraku, Zmasek, Nishimura, & Katoh, 2013).

Individual genes and morphological matrices were concatenated using Sequence Matrix (Vaidya, Lohman, & Meier, 2011). A total of two nested datasets were compiled based on the available information gathered during the analysis, a molecular dataset (MDS), which included 26 taxa exclusively represented by molecular data, and a combined dataset (CDS), which included the same 26 taxa from the MDS combined with 44 morphological characters for each taxon.

### 2.7 Phylogenetic analyses

Individual gene datasets, 18S rDNA, 28S rDNA, 16S rDNA, and COI, as well as all concatenated gene datasets were analyzed using maximum likelihood (ML) and Bayesian methods (BA).

Maximum likelihood (ML) analyses were computed using RaxML version 7.2.8 (Stamatakis, 2006) as implemented on the CIPRES Science Gateway (Miller, Pfeiffer, & Schwartz, 2010). General time reversible (GTR) model of sequence evolution with corrections for discrete gamma distribution (GTR + Γ) was specified for each partitioned dataset, as this is the only model for molecular evolution available in RaxML. Morphological partitions were analyzed using a Markov model (Lewis, 2001). Non‐parametric bootstrapping with 1,000 replicates was used to generate nodal support estimations (Felsenstein, 1985).

Bayesian analyses (BA) were performed using MrBayes version 3.2.5 (Ronquist & Huelsenbeck, 2003) as implemented on the CIPRES Gateway (Miller et al., 2010). Prior to analyses, jModelTest (Posada, 2008) was used for all multiple sequence alignments (MSA) of individual genes to infer their optimal evolutionary model estimated by the corrected Akaike information criterion (AICc). The models selected for each gene included a GTR model with gamma distribution and a proportion of invariable sites (GTR + I + Γ) for 18S rDNA and 28S rDNA. 16S rDNA was run using a GTR + Γ model, and for COI, a Hasegawa–Kishino–Yano model with gamma distribution and a proportion of invariable sites (HKY + I + Γ) was implemented. The morphological partition was analyzed using a Mk1 model (Lewis, 2001). All individual gene datasets and concatenated MDS and CDS were run with two independent analyses using four chains (three heated and one cold). Number of generations was set to 30 million, sampling every 1,000 generations. Burn‐in was set to 10 million generations. TRACER version 1.6.0 (Rambaut & Drummond, 2007) was used to verify convergence of all the MCMC runs. Majority‐rule consensus trees (50%), posterior probabilities, and branch lengths were constructed with the remaining trees after burn‐in.

### 2.8 Species delineation

As mentioned above in *Taxon selection*, species identification of terminals from the same region with similar (or lacking) morphological traits were investigated using species delineation tests. Delineations were made on those terminals of putative similar identification using ultrametric trees obtained in BEAST (see below). Only those terminals corresponding to *Pisione* were utilized in the two most commonly used methods of species delineation (Fontaneto, Flot, & Tang, 2015): the generalized mixed Yule coalescent model (GMYC) (Fujisawa & Barraclough, 2013), and the Poisson tree process (PTP) (Zhang, Kapli, Pavlidis, & Stamatakis, 2013). For all methods, outgroups were excluded from the analyses.

### 2.9 Character evolution tracing

Character evolution was traced using parsimony methods computed with Mesquite version 3.02 (Maddison & Maddison, 2007) and MacClade version 4.0 (Maddison & Maddison, 2000). Most parsimonious reconstructions (MPR) were computed on the tree recovered from the BA of the CDS. MPR, when required, were compared using both the accelerated transformation parsimony model (ACCTRAN) and delayed transformation parsimony model (DELTRAN) available in MacClade.

### 2.10 Testing relative consistency of non‐reproductive versus sexual characters

Most studies on *Pisione* have suggested that the number and structure of male copulatory organs relative to other non‐reproductive character traits show more systematic importance, providing the best basis for morphological species identification. However, a recent review by Salcedo et al. (2015) indicated that certain chaetal and parapodial features should be equally considered. All morphological characters showed a high degree of homoplasy in our character tracing (see results), supporting our decision to test whether, as previously suggested, more emphasis should be placed on copulatory structures than other character traits when performing systematic studies of *Pisione*. We compared the overall consistency index of all the sexual and non‐reproductive morphological characters included in our analyses that exhibit variations within *Pisione*. All sexual and non‐reproductive characters scored by Yamanishi (1998) and Salcedo et al. (2015) pertinent to our samples were included in our morphological matrix, including diagnostic characters for the genus. Consistency indices are widely used to measure the degree of homoplasy in discrete binary characters (Kitching, 1998). A low consistency index indicates a high level of homoplasy, and therefore a large degree of interspecific variation, desirable for species discrimination. Consistency index was calculated as the number of steps for each character divided by the maximum number of steps in the tree from our combined tree using Mesquite version 3.02 (Maddison & Maddison, 2007). A generalized linear model (GLM) was used to investigate the effect of sexual versus non‐reproductive as explanatory characters by the number of steps. The number of steps is considered as discrete counting of the data, for which a Poisson distribution was used as the candidate to fit the model. Boxplots were plotted, and the *z*‐scores generated by the GLM were used to help with the interpretation.

### 2.11 Evolution of copulatory organs

Increasing complexity of copulatory organs within the evolution of *Pisione* has been previously proposed (see Salcedo et al., 2015; Yamanishi, 1998) based on the presence of different morphotypes in different geographical areas. While our dataset does not include all known species of *Pisione,* it does offer an adequate framework to assess the evolution of complexity of copulatory organs. Hence, we tested whether the changes in complexity and number of copulatory organs in *Pisione* were better explained by body size (as in other groups); better explained based on geographical region as proposed by Yamanishi (1998); or, in opposition, phylogenetically constrained. The number of copulatory organs per species was coded as the maximum number of copulatory segments for each species, while copulatory organ complexity was characterized by the number of accessory structures present for each of the species (characters 37–44; Table 3). We characterized body size as the maximum body length and the maximum number of segments. Geographical areas were coded as Western Atlantic, Eastern Atlantic, and Indo‐Pacific, according to Yamanishi's criteria. Checks for autocorrelation of continuous characters were performed before submitting them for further analyses. Ultrametric trees calculated with BEAST version 1.8.3 (Drummond, Suchard, Xie, & Rambaut, 2012) and all available terminals were used as a framework to evaluate the evolution of male copulatory structures in *Pisione*. These character trees only included those terminals from previous analyses that represented different species. BEAUTi version 1.8.3 was used to generate all the xml files for the BEAST runs. GTR + Γ models were selected for the 18S rDNA and 28S rDNA partitions, 16S rDNA was run using a GTR, and for COI, a HKY + I + Γ was implemented. Tree priors were selected under a Yule process. Analyses were run with independent MCMC chains, which were set for 100 million generations. Sampling was set every 10,000 generations. Convergence of the reads was confirmed using Tracer version 1.5 (Rambaut & Drummond, 2007). The consensus tree based on maximum clade credibility (MCC) was obtained using TreeAnnotator version 1.6.1 with a burn‐in of 20% (Drummond et al., 2012).

The hypothesis that number and complexity of copulatory organs are phylogenetically constrained was evaluated using Pagel's lambda (λ) and Blomberg's *K* indices with the function “phylosig” as implemented in the R package phylotools (Revell, 2012). Values close to 1 provide a high indication that the characters are phylogenetically constrained, whereas values near zero indicate that the character is highly variable between closely related species.

Alternatively, we investigated whether there was evidence of coevolution of both body size and the number and complexity of copulatory organs, while taking into account the effect of geographical areas. This was evaluated using the phylogenetic general least square methods (PGLS). The logarithm of the maximum number of copulatory structures and copulatory complexity were the response variables, treated as continuous, with a Gaussian distribution. The logarithm of maximum body length, the logarithm of the maximum number of segments, and the geographical area were selected as explanatory variables. All models were investigated using Monte Carlo Markov chains within the package MCMCglmm (Hadfield, 2010), which was run for 5.1 million generations. The first 100,000 generations were discarded as burn‐in, and the thinning parameter was set to 500.

The evolution of maximum number of copulatory organs, copulatory organ complexity, maximum body length, and the maximum number of segments was estimated on the obtained ultrametric tree to visually illustrate our results. Each continuous character was traced using the function contMap (Revell, 2013) implemented in the R package phylotools (Revell, 2012).

### 2.12 Analyses of geographic distribution patterns

Geographical distribution patterns were analyzed from geographic collection data for all 55 described and unidentified species of *Pisione*. Distribution of the species was plotted with the package “maps” and “mapdata” in R (Becker, Wilks, Brownrigg, & Minka, 2013; Brownrigg, 2013).

Latitude preferences for *Pisione* were inferred for tropical, subtropical, and temperate zones using the correlation between the numbers of species recorded at each 20 degrees of latitude in order to predict if there is a latitudinal gradient (LDG), where, *for example,* the biological diversity is increasing from the poles to the tropics (Jablonski et al., 2006). We also predicted that *Pisione* diversity is correlated to the Caribbean and Indo‐Pacific Oceans, usually interpreted as biogeographical hotspots for several metazoan groups (Bowen et al., 2013).

We counted the number of species for latitude in steps of circa 10 degrees. Our first set of ranges started at 0 degrees to 9.9, from 10.0 to 19.9, from 20.0 to 29.9, etc., in the same manner, and following the same approach for the remaining latitudes. This was repeated and also used for the negative latitudes. *Pisione* preferences for the Indian Ocean, Pacific Ocean, and the Atlantic Ocean were tested using the correlation between the numbers of species recorded in each longitudinal subgroup every 40° of longitude following similar approaches to those for the latitude. The maximum diversity of *Pisione* was used to infer latitudinal and longitudinal preferences. Geographical ranges with maximum diversity were estimated using spline models (Anderson, 2008; Di Domenico, Martínez, Lana, & Worsaae, 2014; Koenker, 2005; Koenker, Ng, & Portnoy, 1994) built for the 95th percentile. Models were fitted using the functions rq() and bs() [“quantreg” package for R; (Koenker, 2007; R Core Team, 2014)]. The degree of the polynomial was chosen by the AICc corrected for small samples (Burnham & Anderson, 2003; Hurvich & Tsai, 1989). The model with the smallest AICc value from a set of models with a degree of polynomial was selected, and optimal values were interpreted. Ninety‐five percent bootstrap confidence intervals (Manly, 2006) were obtained for each of the 10k bootstrapped sample pairs using the polynomial degrees that were chosen for the original data.

## 3 Results

### 3.1 Phylogenetic analyses

**Figure 3:**
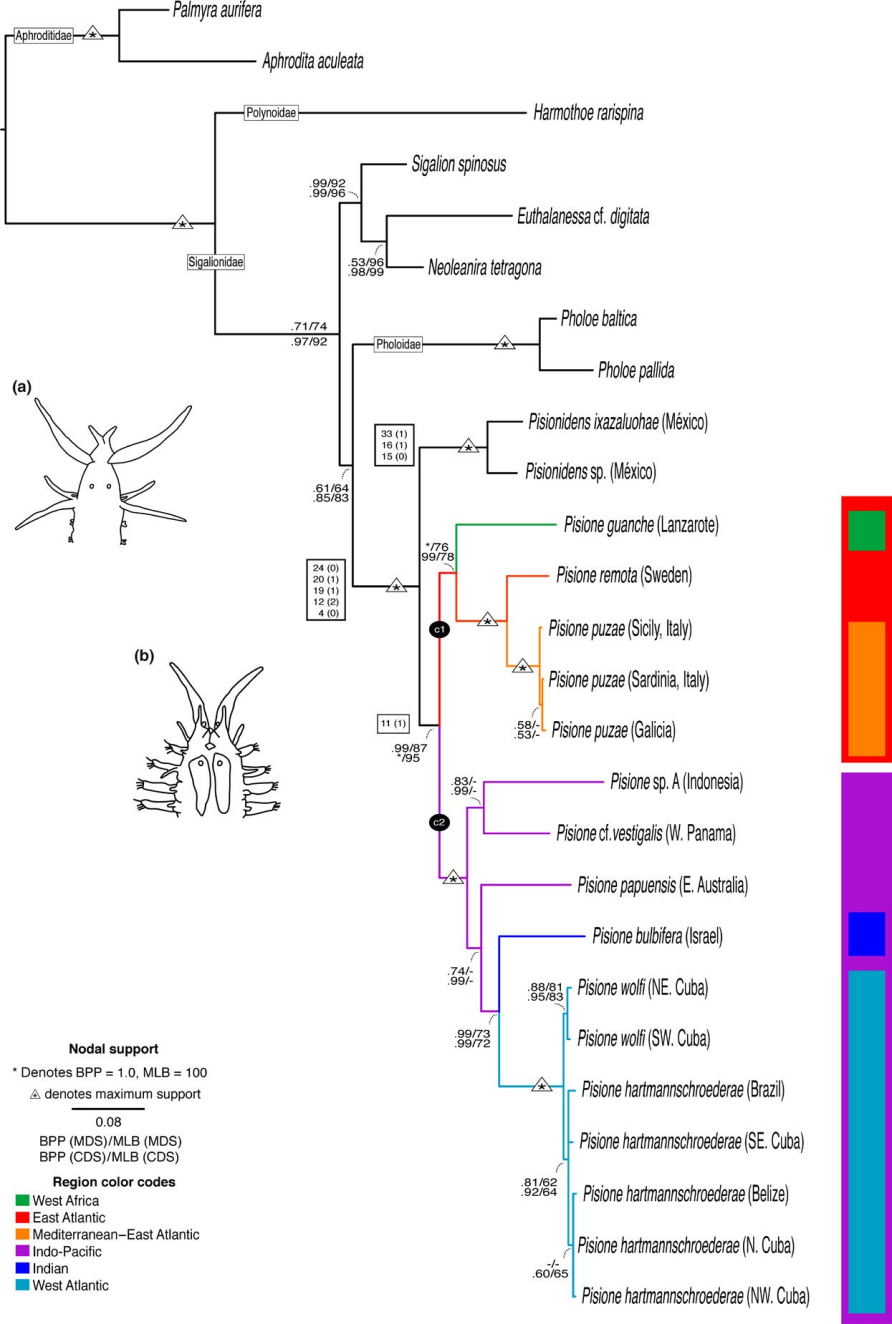
Biogeographical regions within *Pisione*. Tree topology based on the Bayesian analysis (BA) of the combined dataset. Only nodal support above BPP = .5 and MLB = 50 is displayed. Nodes not recovered or with low support are illustrated with a dash (–). Triangles with asterisks inside represent maximum support in all analyses, while a single asterisk (*) denotes maximum support in a specific analysis (BPP = 1.0 or MLB = 100). Boxes on branches identify apomorphies, including character number and states in parentheses. Full character coding for all terminals can be found in Table 2. (a) Schematic representation of *Pisionidens*, including prostomial appendages and first few segments (redrawn from Rouse & Pleijel, 2001). (b) Diagrammatic representation of *Pisione*, including prostomial appendages and first few segments (redrawn from Rouse & Pleijel, 2001)

The Bayesian and maximum likelihood analyses recovered similar topologies between the MDS and CDS analyses and are presented in Fig. 3 with both Bayesian posterior probabilities (BPP) and maximum likelihood bootstrapping (MLB) values. However, between the methods, slight differences were recovered and are described below.

The terminals representing the genera *Pisione* and *Pisionidens* formed reciprocally monophyletic clades, both with high support (Fig. 3). *Pisione* comprised two clades associated with geographic regions that we have designated as clade‐1 (c1) consisting of the Eastern Atlantic clade *Pisione guanche – Pisione puzae* (CDS: BPP .99; MLB 78), and clade‐2 (c2) consisting of the clade *Pisione *cf. *vestigalis* – *Pisione hartmannschroederae* (CDS: BPP 1.0; MLB 100), representing distributions across the Western Atlantic and Indo‐Pacific regions (Fig. 3).

Clade‐1 consisted of the Eastern Atlantic species including *P. guanche*,* Pisione remota* (Southern 1914), and *P. puzae* Siewing, 1953, represented by specimens collected in Napoli, Sardinia (Italy), and Galicia (NW Spain). These species branched off sequentially from the root, all with comparatively well‐supported relationships with identical topologies regardless of the method (Fig. 3).

Clade‐2 always recovered a fully supported Indo‐Pacific clade and West Atlantic subclade regardless of the method. However, relationships between some of the species within clade‐2 were less stable and varied depending on the methods. This was due to the varying position of *Pisione papuensis* Govaere & De Wilde, 1993 which was not supported in MLB (CDS: MLB < 50). BA recovered the taxa *P. *cf. *vestigalis* and *Pisione* sp. A (Indonesia) in a sister relationship (CDS: BPP .99), however MLB < 50. These two taxa are sister to a clade (CDS: BPP .99) that includes *Pisione bulbifera* Yamanishi, 1998 and subclade *Pisione wolfi – Pisione hartmannschroederae,* represented by specimens collected from several localities (CDS: BPP .99; MLB 72). Those taxa collected from the West Atlantic region, which included Brazil, and Cuba and Belize from the Caribbean, formed a fully supported clade in all analyses. Different sequences of *P. wolfi*, collected throughout Cuba, formed a subclade (CDS: .95; MLB 83), which was sister to a subclade with several populations of *P*. *hartmannschroederae* Westheide, 1995 (CDS: BPP .92; MLB 64). All morphological species represented by more than one specimen were found monophyletic with respect to other species of *Pisione*.

### 3.2 Species delineation

In the absence of diagnostic morphological penile traits or in cases of damaged specimens, species delineation tests made it possible to distinguish the least taxonomic units. While pairwise genetic distances may resolve such issues, we preferred to implement more accurate methods given the high interspecies variability within *Pisione* (Fontaneto et al., 2015). Species delineation techniques employed recovered a single species representing the Mediterranean–East Atlantic region, *P. puzae,* with representatives from both Italy and Galicia. In the West Atlantic region, *P. wolfi* was found throughout the Cuban coastline, and *P. hartmannschroederae was* recovered with large distributions throughout the West Atlantic.

### 3.3 Character evolution tracing and analyses

The relationship of the members within the clade *Pisionidens* – *Pisione* was supported by five synapomorphies, traced without homoplasy (see Appendix 1). These included the absence of a median antenna (character 4), the position of dorsal cirri on all segments (character 13), modified stylode with papillated/adhesive disks (character 19), parapodia modified for reproduction (character 20), and the absence of notochaetae (character 24).

The *Pisionidens* clade was supported by three synapomorphies, the lack of biramous parapodia (character 15), lateral glandular fields (character 16), and the presence of midventral pores (character 31). The only synapomorphy supporting the *Pisione* clade and subclades within was the presence of buccal aciculae (character 11). The remaining characters coded for the ingroup were traced with homoplasy. The non‐reproductive character 32 (presence of protruding notoaciculae) and the sexual characters 37 (fusion of copulatory organ and parapodial lobe), 41 (presence of sheath‐like arc of the copulatory organ), and 44 (presence of spinous papillae on copulatory structures) were all traced with one step. The non‐reproductive character presence of long‐bladed compound chaetae (character 36) and the sexual character presence of bidigitate process of copulatory organ homologous to inferior stem (character 40) were traced with two and three steps, respectively, supporting the clades *Pisione papuensis – Pisione hartmannschroederae* and *Pisione guanche – Pisione puzae* as well as *Pisione* sp. A – *Pisione *cf. *vestigalis,* respectively. The remaining characters showed even a higher level of homoplasy, traced with more than three steps within *Pisione*.

**Figure 4:**
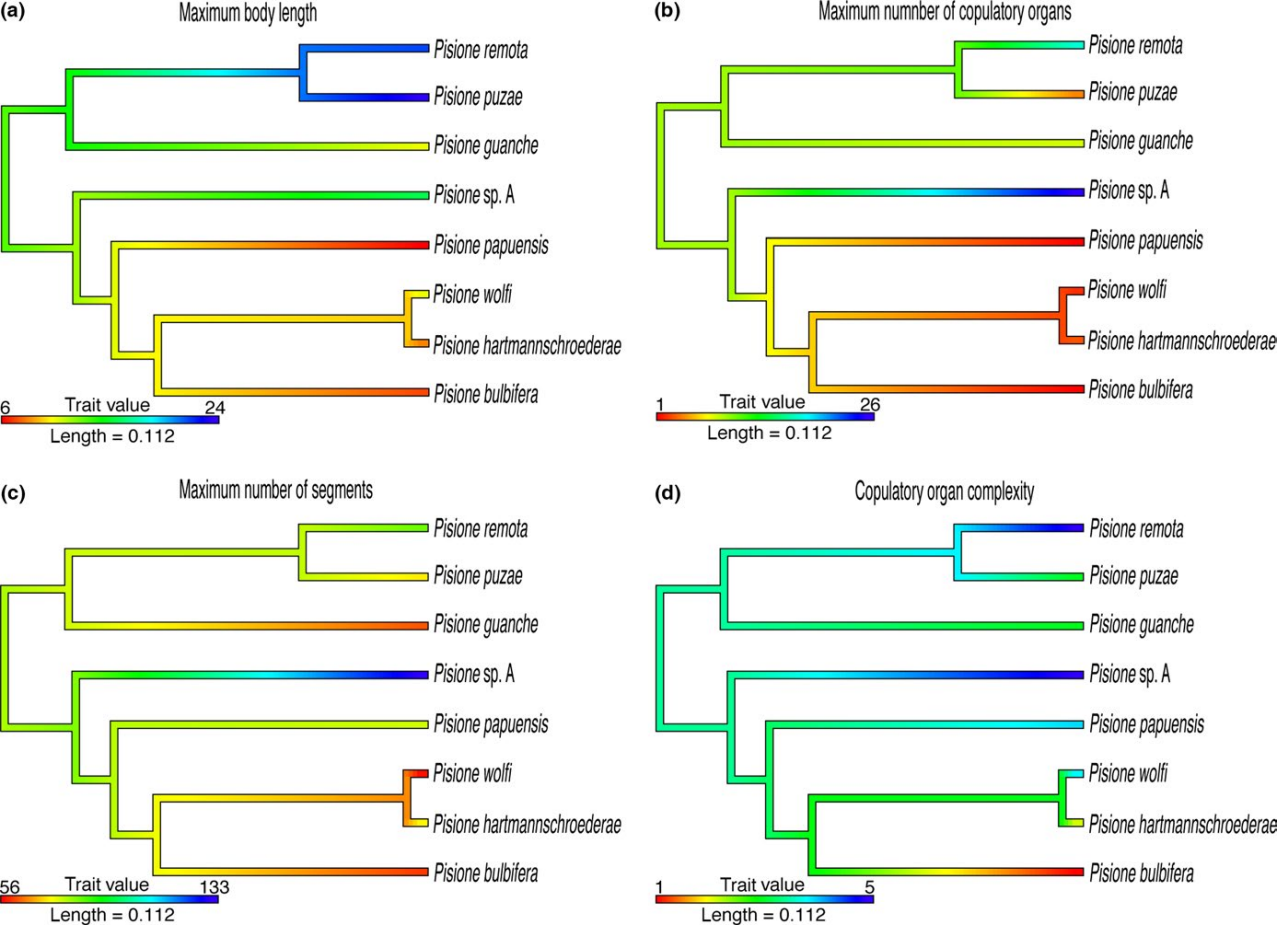
Ultrametric trees (a–d) representing the evolution of continuous characters from species included in the phylogeny. Each continuous character was traced and color‐coded; with different tones correlating to the value of the character for each node. Warm colors (i.e., red and orange) correspond to small values, while cool colors (i.e., blue and green) correspond to large. The correspondence of colors and trait values, as well as ranges of each of the traits is summarized in the individual tree legends: (a) maximum body length. (b) Maximum number of copulatory organs. (c) Maximum number of segments. (d) Copulatory organ complexity

Overall, the level of homoplasy between sexual and non‐reproductive characters was the same, reflected by comparable distribution of the number of steps across both groups of characters (Fig. 4), of which *z*‐scores calculate by the GLM did not yield any significant differences (*z *=* *1.09; *p *=* *.28). Although no differences were observed between sexual and non‐reproductive characters, the intercept was different from zero steps (*z *=* *2.19; *p *=* *.028), indicating some level of homoplasy equally predicted by both characters.

### 3.4 Evolution of copulatory organs

The maximum number of paired copulatory structures and their complexity, estimated as the number of accessory copulatory structures, showed low phylogenetic signal (number of copulatory organs λ = 0.00, *K *=* *0.241; complexity of copulatory organs λ = 0.00; *K *=* *0.252). PGLS was unable to show any relationship between maximum number of copulatory organs, complexity of copulatory organs, or any of the explanatory variables. The number of paired copulatory organs is reduced independently in two clades, including *P. puzae* clade and *Pisione papuensis – Pisione hartmannschroederae,* while increasing in *Pisione* sp. A. The complexity of the copulatory structures is reduced once in *P. bulbifera*, but increases independently toward *P*. *guanche* and *P. vestigialis*.

### 3.5 Geographic distribution patterns

**Figure 5:**
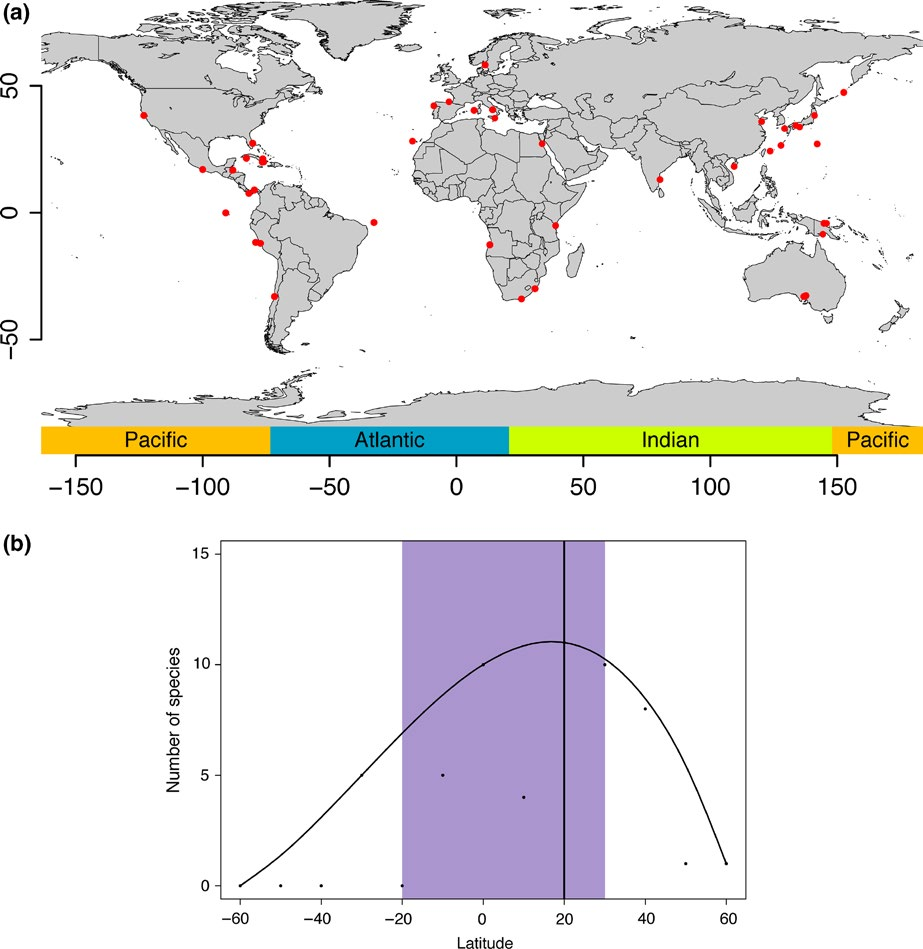
Geographic distribution and analyses of *Pisione* (a–c). (a) Red circles indicate the collection localities of all species of *Pisione*. (b) Spline smoothing with polynomial regression (*n* = 3) of latitude with number of species of *Pisione*. Vertical line demarks the optimum value for diversity, and 95% confidence intervals are shown in shaded box

Geographic analyses yielded a well‐supported diversity gradient of *Pisione*, with a maximal diversity estimated at 20° latitude, with a range between −20° and 30° latitude within a 95% confidence interval. A steep decrease in diversity was present in latitudes >−20° and >30° toward both poles (Fig. 5).

The comparison of the longitudinal distribution patterns showed no significant optimal values and is not illustrated.

## 4 Discussion

### 4.1 Phylogenetic relationships of *Pisione*

*Pisione* was well‐supported, recovered sister to *Pisionidens*, and with buccal aciculae as a morphological synapomorphy. We recovered two clades within *Pisione* supported by comparatively high nodal support values, but lacked identifiable synapomorphies. Comparably, both sexual and non‐reproductive morphological characters were traced with high levels of homoplasy. Similar patterns of character evolution with numerous homoplasious characters have also been found in other lineages of interstitial annelids, including Saccocirridae (Di Domenico et al., 2014), Protodrilidae (Martínez, Di Domenico, Rouse, & Worsaae, 2015), and Nerillidae (Worsaae, 2005, 1883). However, generally in these families, the major internal clades could more easily be diagnosed by unique combinations of morphological characters (Di Domenico et al., 2014; Martínez et al., 2015). In contrast, within each of the recovered *Pisione* clades, nearly all morphological characters were traced with a high degree of homoplasy and reversals within the clades. This makes any empirical diagnosis challenging and pleas for a denser phylogenetic sampling of *Pisione* terminals as well as new morphological examinations, possibly refining the definition of character states.

Intriguingly, both of the clades recovered within *Pisione* were associated with different geographical areas, clade‐1 restricted to the East Atlantic (Mediterranean and the Canary Islands), and clade‐2 distributed along the Indo‐Pacific (Indonesia, Israel) and Western Atlantic (Caribbean and Brazil). Comparable distribution patterns have also been recovered within the interstitial protodrilid genus *Megadrilus* Martínez, Di Domenico, Rouse & Worsaae, 2015; which also showed an East Atlantic and Western Atlantic–Indo‐Pacific clades (Martínez et al., 2015). The similar distribution patterns between *Pisione* and *Megadrilus* might be related to the presence of a common dispersal strategy, as they both are comparatively large species with pelagic larvae. However, these similar distributions may just be due to common vicariant processes or sampling biases that have artificially produced similar distribution patterns.

### 4.2 Character evolution tracing and analyses

Yamanishi (1998) argued that reproductive characters, specifically the male copulatory organs and accessory structures, are the most important morphological diagnostic characters for describing and identifying *Pisione* species. However, given that male sexual maturity is seasonal within *Pisione*, mature males are often lacking from collections. This scenario has complicated descriptions, and those descriptions lacking males usually rely on size comparisons or on extremely divergent characters. Salcedo et al. (2015) argue that additional characters are needed in conjunction with male copulatory structures, especially those that are expressed regardless of sexually maturity or season. These characters should include type and number of neurochaetae, shape of the dorsal cirri on segment three, neuropodial lobes, and shape, size, and ornamentation of the neuro‐ and buccal acicula. While these character transformations rarely constitute apomorphies, they may be of systematic importance when combined, as several species of *Pisione* either lack aciculae (neuro‐ or buccal‐) or may exhibit intraspecific variation within neurochaetal numbers and patterns.

Building atop of these previous ideas, when we compared sexual characters to that of non‐reproductive characters, we find that there are no comparable differences in the degree of homoplasy between them. Both sexual and non‐reproductive characters lack both clade and regional specificity. While the results do not refute Yamanishi (1998), they do add emphasis to the findings of Salcedo et al. (2015) that proper descriptions and identification should take into account both sexual and non‐reproductive characters. This is in agreement with the most recent investigations on interstitial annelids from other groups, including Nerillidae (Worsaae, Martínez, & Núñez, 2009; Worsaae & Rouse, 2010), Protodrilidae (Di Domenico, Martínez, da Cunha Lana, & Worsaae, 2013; Martínez et al., 2015), Psammodrilidae (Worsaae, Kvindebjerg, & Martínez, 2015; Worsaae & Sterrer, 2006), and Saccocirridae (Di Domenico et al., 2014; Jouin‐Toulmond & Gambi, 2007), which have shown that while both sexual and non‐reproductive characters are needed for species identification, so is the implementation of various microscopy techniques to distinguish both external and internal variation. Furthermore, given the degree of homoplasy across morphological characters, it suggests that multiple specimens should be investigated and discussed in order to account for intraspecific variation due to varying degrees of maturity in male species. Unfortunately, *Pisione* are often found only in low numbers or broken during the extraction process, further compounding the issue and hindering observations of intraspecific variation.

### 4.3 Evolution of copulatory organs

While we have shown that sexual characters are equally important as non‐reproductive, this is the first comparative investigation into the organization and complexity of copulatory structures in *Pisione* based on comparative phylogenetic methods. While attempting to address both species level identification and evolutionary and biogeographical patterns, Yamanishi (1998) explained that it was possible to group species based on their degree of complexity in male copulatory structures. While morphological phylogenetic analyses were not performed by Yamanishi (1998), five groups based on copulatory complexity were illustrated (Yamanishi, 1998; fig. 22), providing a framework for our current comparative investigations. Three of the illustrated groups were proposed to represent an evolutionary succession of increasing complexity (“africana” → “remota” → “crassa”); however, there were two groups that were characterized separately: a “papuensis” group that shared mixed similarities of the “africana” and “remota” groups and may potentially represent a hybrid or intermediate group, and a “gopalai” group that is highly modified and unlike the other groups, with fusion of several characters and lacking any protruding structures. While biogeographical patterns were not emphasized by Yamanishi (1998), he willfully excluded several of the known species at that time from his construction of his copulatory complexity progression scheme, including several members from within the Western Atlantic (including the Caribbean).

Present study included representatives of the proposed successive “africana” (*P. guanche*), “remota” (*P. remota, P. puzae*), and “crassa” (*P. *cf. *vestigialis*) groups, all having an elongated copulatory organ stem, as well as the “papuensis” (*P. papuensis*) group, with stem of copulatory organ broad and partially fused to the parapodia. Our comparative analyses reject the evolutionary trend of both an increase in number of copulatory organs and an increase in complexity of copulatory structures across represented species of *Pisione* (Fig. 3). Within the phylogeny, there were multiple instances of both increasing and reduction of complexity and number of copulatory structures. More significantly, our phylogenetic character tracing revealed a high degree of variability and homoplasy in the characters associated with copulatory and accessory structures, reflected by the paraphyly of, at least, two of Yamanishi's copulatory groups (i.e., “crassa” and “remota” groups). Taking into account the limitation of our dataset, our results suggest that it is highly unlikely that even the addition of more taxa will change the general findings to support Yamanishi's theory of increasing complexity. Furthermore, when we consider our ancestral character reconstructions, the *Pisione* ancestor was estimated to bear an intermediate number of copulatory organs with a medium complexity of accessory structures. This is in direct opposition to Yamanishi's (1998) proposal that the ancestral state would represent a large bodied *Pisione* with simple male copulatory structure resembling more closely that of the “africana” group.

The evolution of copulatory organs within *Pisione* appears highly convoluted. Within our East Atlantic clade‐1, *P. guanche* exhibits copulatory structures similar in form to that of the “africana” group, having an elongated copulatory organ proper, lacking whorls, with elongated ventral cirrus with unmodified neurochaetae (Martín et al., 1999; Yamanishi, 1998). In our analyses, this so‐called primitive group (according to Yamanishi, 1998) was recovered as the sister taxon to *Pisione remota – Pisione puzae clade*. This clade displays copulatory organs related to the “remota” group, which bears a bidigitate process on the main stem of the copulatory organ proper (Yamanishi, 1998). When we investigated the morphology with both SEM and that of descriptions by Yamanishi (1998), a slight evolutionary progression similar to what was originally proposed by Yamanishi can be seen, especially when comparing *P. guanche* to *Pisione remota – Pisione puzae*. However, these three species are not closely related to *P. vestigialis* or *P. wolfi,* the two species included in our analyses belonging to the “crassa” group, which display highly ornamented copulatory structures. Furthermore, copulatory structures associated with the “remota” group (i.e., *P. remota* and *P*. *puzae*) are known to display additional accessory structures not present in the “africana” group. However, our character reconstructions show that the homoplasious characters 42–44 are present in our *Pisione* representatives from both the “africana” and “remota” groups. Additional shared characters (45–46) present in the *Pisione remota – Pisione puzae* clade (“remota” group) are also found in terminals from clade‐2 that further invalidate Yamanishi's assumptions of the “remota” group being of single origin from the “africana” group. In the Indo‐Pacific–West Atlantic (clade‐2), species of *Pisione* exhibited copulatory structures indicative of the “papuensis” group, the “crassa” group, and that of the “remota” group. *Pisione* cf. *vestigalis* and *P. wolfi* both share copulatory structures resembling that of the “crassa” group; however, these species are each nested within separate subclades and geographical regions, respectively, hereby also negating the monophyly of Yamanishi's “crassa” group. *Pisione papuensis* is found to be the sister taxon of a clade containing *P. bulbifera*, that in itself bears copulatory structures resembling the “remota” group. The close association of both the “papuensis” and “remota” groups is not surprising given the similarly enlarged copulatory organ proper and ventral cirri, contributing to close morphological affinities. Yet, again, the close association of these species breaks up the linear evolutionary scenario of Yamanishi (Fig. 2a), which is further compromised by the two unrelated origins of the “crassa” forms (*P*. cf. *vestigalis* and *Pisione wolfi – Pisione hartmannschroederae*) within clade‐2. This varying morphology in our analyses seen by our recovered clades (Fig. 3) is attributed to several homoplasious characters observed during multiple species examination with detailed microscopy. It appears that the more complex evolutionary scenario found herein may already have been suspected by Yamanishi, who himself excluded several species from his classification of male copulatory structures.

### 4.4 Distribution patterns

Outside of taxonomical descriptions, no other study attempts to describe biogeographical patterns within *Pisione*. In an attempt to understand patterns of diversity, Yamanishi (1998) was the first to address the widespread distribution ranges and diversity in Japan. Several species or morphotypes within *Pisione* (i.e., *Pisione africana* Day, 1963; *Pisione gopalai* (Alikunhi, 1941); *Pisione parva* De Wilde & Govaere, 1995) are capable of wide range dispersals, across both hemispheres and broad temperature ranges, with only minute changes in morphology observed at the subspecies level (Yamanishi, 1998). Aside from the more limited transfer by storms or other migrating sand events, these wide range dispersal events may be due in part to a relatively long planktonic stage and/or perhaps dispersal by anthropogenic means. *Pisione* larvae drift about the pelagic, capable of collecting food by mucoid or slime nets, and can last up to 10 days in search of suitable substrate (Åkesson, 1961). The patterns of oceanic currents coupled with long planktonic larval stages suggest this as the most plausible means of dispersal within *Pisione*. While many other dispersal vectors have been proposed for additional groups of interstitial annelids (see Weidhase, Bleidorn, & Simon, 2016), ballast sand or water may also play a role in the dispersal of *Pisione* individuals.

Our analyses revealed two large clades within *Pisione*, one that appears to be restricted to the East Atlantic, including the Mediterranean, and the one spanning from the Red Sea, across the Pacific, and into the West Atlantic including the Caribbean. To date, *Pisione* has always been described as being most commonly distributed within tropical and subtropical areas (Aguado & San Martín, 2004; Salcedo et al., 2015; Yamanishi, 1998). Our geographic analyses confirm this statement, showing maximum diversity across the latitudes −20° to 30° (Fig. 4). We considered that the number of sampling efforts and records might generate false‐positive correlations between diversity, and that of latitude and longitude preferences; however, we attempted to address this issue by using a confidence interval for the optimal value (peak of the unimodal curve, 20°) to fit the curve. This method, albeit exploratory, appears to have mitigated our concerns, as the historically high sampling efforts in European waters were not displayed as the highest estimates of diversity, nullifying any false‐positive correlation. Furthermore, over half of the described species, including all of the subspecies, are reported from within the West Pacific and Indian Oceans (Salcedo et al., 2015). The effect of area in regard to high diversity at lower latitudes (tropics) has been historically debated in ecology. Given our distribution patterns (Fig. 5) of species richness within *Pisione,* at first glance it appears to possibly resemble that of the so‐called mid‐domain effect [MDE; a controversial null model that generates patterns by random overlap of geographic ranges in low latitudes (Colwell & Lees, 2000; Hawkins, Diniz‐Filho, & Weis, 2005)]. While addressing this issue was not the focus of this manuscript, our implementation of the bootstrap (implemented when estimating the optimum peak) provides an additional tool to assess the uncertainty of our estimates and our sampling efforts (Kulesa, Krzywinski, Blainey, & Altman, 2015). While we agree that this method lacks a traditional statistical inference, it may represent a more rational way to find and fit models than the traditional linear predictive models that are questionable when it comes to low sampling efforts and area effects. Unfortunately, obtaining significant optimal values in our longitudinal distribution patterns was not possible.

The overall pattern within the terminal clade of West Atlantic (Caribbean) species being most closely related to Indo‐Pacific taxa, while speculative herein, is a well‐known pattern among tropical marine fishes, and may be associated with connectivity through the Panama Gateway until ca. 4.5–3.0 Mya (Bartoli et al., 2005; Haug & Tiedemann, 1998). While the East Pacific and West Atlantic tropical marine fauna generally has a history of isolation after the closure of the Panama Gateway and thereby less diversity than in the Indo‐West Pacific (Cowman & Bellwood, 2013), a few taxa have had a profound recent radiation in the East Pacific and the Atlantic Caribbean, *for example* wrasses (Labridae) (Barber & Bellwood, 2005). Our results from Cuba indicate that a similar profound radiation is ongoing for *Pisione* in the West Atlantic. An alternative hypothesis is that the West Atlantic tropical fauna is a relictual assemblage of a western Tethyan fauna isolated by the closure of the Red Sea Land Bridge at 18–15 Mya (Steininger & Rögl, 1984) as shown for corals (Budd, 2000). However, lack of fossils for calibration prevents aging of *Pisione* clades, and the observed distribution patterns and diversity may very likely just reflect sampling bias in this first phylogeny of *Pisione*.

## 5 Conclusions

Our phylogenetic analyses revealed a well‐supported monophyletic *Pisione* with subclades representing biogeographical regions. While to date this is the largest collection of molecular data for the interstitial *Pisione,* it marks only the first step toward understanding the evolution of copulatory organs within. Our character analysis revealed that both reproductive and non‐reproductive morphological characters are highly homoplasious and are not informative as stand‐alone characters. As a consequence, our character tracing only revealed a single synapomorphy defining *Pisione*, with no single character useful toward biogeographical and/or phylogenetic patterns of the subclades within *Pisione*. Additionally, species delineation techniques revealed large dispersal ranges, especially for *P. hartmannschroederae* in the West Atlantic, similar to what Yamanishi claimed for some *Pisone* species from the Pacific. Given our phylogenetic results, we no longer find a basis to support Yamanishi's concept of increasing complexity within male copulatory organs. Instead, it appears that within *Pisione,* evolution toward less complex and more copulatory organs has taken place. Additionally, our ancestral character reconstruction suggests that the ancestral organization of the male copulatory organ would be of an intermediate form, and not one of simplicity as Yamanishi proposed. While our findings continue to show that structures of male copulatory organs are uniquely species specific, questions still remain regarding the need for such specialization, specifically, what effect ecological and or geographical parameters may have on male copulatory variation.

## Conflict of Interest

All authors declare that they have no competing interests.

## Appendix 1 Character coding states and descriptions

### 1.1

Detailed descriptions of all 44 morphological characters, including character states and references. Coding of individual taxa used in this study is presented in Table 3.

### 1.2

1. *Palp surface [1]: smooth [=0]; papillose [=1]; ciliated [=2]; rugose [=3]*: Palp surface texture is highly variable in scale worms. Within this study, members of Sigalionidae exhibit palps that are smooth (e.g., Pettibone, 1992), papillose, or rugose (wrinkled or corrugated‐like, this study). All *Pisione* have been coded as having rugose palps. Aphroditidae and Polynoidae taxa of outgroups exhibit papillose palps (e.g., Barnich & Fiege, 2009; Pettibone, 1986), and *Palmyra aurifera* has ciliated palps (Watson Russell, 1989).
2. *Inner palpal sheath [2]: absent [=0]; present [=1]*: Medial continuation of anterior margin of segment one (e.g., Govaere & De Wilde, 1993; Pettibone, 1970, 1997). Presence restricted in this study to several genera of Sigalionidae, including all species of *Pisione* incorporated in this study. Absent in *Pisionidens* and outgroup taxa of Aphroditidae and Polynoidae.
3. *Outer palpal sheath [3]: absent [=0]; present [=1]*: Lateral continuation of the anterior margin of segment one (e.g., Pettibone, 1970). Presence restricted in this study to members of Sigalionidae including most *Pisione* taxa. Absent in *Pisionidens* and outgroup taxa of Aphroditidae and Polynoidae.
4. *Median antenna [4]: absent [=0]; present [=1]*: A median antenna is absent in all taxa within the interstitial Sigalionidae genera *Pisione* and *Pisionidens*. Variable in outgroup taxa, including within Sigalionidae.
5. *Lateral antennae [5]: absent [=0]; present [=1]*: Lateral antenna are absent in all *Pisione* taxa. *Pisionidens* have frontal projections termed antenna, which have been scored in this study as lateral antennae. Presence of lateral antennae varies within outgroup taxa.
6. *Prostomial shape [6]: subrectangular [=0]; oval [=1]*: The shape of the most distal margin of the prostomium is subrectangular (e.g., Barnich & Fiege, 2009) or is completely rounded (e.g., Naeini & Rahimian, 2009). All *Pisione* are coded as being subrectangular. Outgroups are with both states.
7. *Prostomium [7]: without lobes [=0]; bilobed [=1]*: The presence of a median groove divides the prostomium into distinct lobes (e.g., Pettibone, 1985). Variable in *Pisionidens* but bilobed in all *Pisione* taxa. Outgroup taxa variable.
8. *Tentacular cirri [8]: absent [=0]; present [=1]*: Tentacular cirri of segment one are present in all *Pisione* but absent in *Pisionidens*. Present in outgroups.
9. *Eyes [9]: absent [=0]; present [=1]*: Presence of paired eyes (usually two pairs) is variable in scale worms. All *Pisione* coded as having eyes. Present in all outgroup taxa except *Neoleanira tetragona* and *Pholoe pallida*.
10. *Segment 1 [10]: achaetous [=0]; bearing chaetae [=1]*: Segment 1 may lack chaetae or exhibit single (e.g., Barnich & Fiege, 2009), or multiple chaetae (e.g., Pettibone, 1963). In all *Pisione* and *Pisionidens* segment one is achaetous. Variable in outgroup taxa.
11. *Buccal aciculae [11]: absent [=0]; present [=1]*: Aciculae of segment 1 are typically protruding in *Pisione,* proximal to the base of each palp. Presence limited to *Pisione* taxa. Absent in *Pisionidens* and all outgroup taxa.
12. *Notopodial sensory projection [12]: absent [=0]; present as dorsal cirri [=1]*: Notopodial sensory projections are cirriform structures derived from the superior notopodium (Mikkelsen & Virnstein, 1982) with styles tapered distally. Present in *Pisione* and *Pisionidens*. Variable within outgroups.
13. *Position/distribution of the dorsal cirri [13]: only on segment 3 [=0]; on most non‐elytrigerous segments [=1]; on all segments [=2]*:* Pisione* and *Pisionidens* have dorsal cirri on all segments. Various arrangements within the non‐elytrigerous segments of outgroup taxa.
14. *Dorsal tubercles on non‐elytrigerous segments [14]: inconspicuous [=0]; prominent [=1]*: In Aphroditiformia, tubercles may be positioned on non‐elytrigerous segments in line with elytrophores. Absent in *Pisione* and *Pisionidens*. Variable throughout outgroup taxa.
15. *Parapodial form [15]: all uniramous [=0]; biramous from segment 2 [=1]*: Only *Pisionidens* (Aiyar, 1943) exhibits true uniramous parapodia and lacking notoacicula. All remaining *Pisione* taxa have biramous parapodia with the notopodia represented only by the presence of notoacicula from segment 2. Outgroup taxa are all biramous from segment 2.
16. *Lateral glandular fields [16]: absent [=0]; present [=1]*: Small grouping of pores/glands present between parapodia along body wall (Petersen et al., 2016). Present in *Pisionidens ixazaluohae* and unknown for *Pisionidens* sp. Absent in all other taxa.
17. *Notopodial stylodes [17]: absent [=0]; present [=1]*: Finger‐like projections scattered on surfaces of the notopodia (e.g., Chambers, 1985). The character is absent in *Pisione*. Present only in outgroups.
18. *Neuropodial stylodes [18]: absent [=0]; present [=1]*: Finger‐like projections scattered along the neuropodia in certain Sigalionidae including *Pisionidens* and *Pisione* (e.g., Govaere & De Wilde, 1993). The feature is variable in outgroup taxa.
19. *Modified stylode with papillated/adhesive disks [19]: absent [=0]; present [=1]*: Numerous individually papillated/adhesive disks present on a large, non‐protruding stylode, at the most distal part of the neuropodia. The function of these structures is unknown but may serve for adhesion, as sensory structures, or a combination of the two. Only present in *Pisione* and *Pisionidens*. The feature is absent in outgroup taxa with neuropodial stylodes, and scored as inapplicable for those outgroup taxa that lack neuropodial stylodes.
20. *Parapodia modified for reproduction [20]: absent [=0]; present [=1]*: Presence of a single pair (per segment) of male copulatory organs are found in *Pisione* and *Pisionidens* (e.g., Day, 1967; Martín et al., 1999). The number and ornamentation of paired male copulatory segments vary with taxa. Copulatory segments lack dorsal cirri; however, an elongation and/or modification of ventral cirri occurs in some taxa of *Pisione*. Terminals with only female specimens were coded as present due to separate sexes observed in both *Pisione* and *Pisionidens*. Copulatory organs not present in outgroup taxa.
21. *Proboscis/muscular pharynx [21]: without chitinized structures [=0]; with chitinized structures [=1]*: A muscular axial proboscis is present in all Aphroditiformia taxa (Rouse & Pleijel, 2001) and is usually accompanied by opposable chitinized structures. These structures are absent in some outgroup taxa.
22. *Number of segments [22]: ≤50 [=0]; ≥51 [=1]*: Sigalionidae taxa typically all have segments numbering greater than 51, but interspecific variability may occur. Variable in outgroup taxa.
23. *Prechaetal lobes [23]: absent [=0]; present [=1]*: Small lobes present in front of protruding chaetae. Variable within *Pisione* but an important character for identification of some species. Absent in all outgroup taxa.
24. *Notochaetae [24]: absent [=0]; present [=1]*: Notopodial chaetae are absent in *Pisione* and *Pisionidens*. Present in all outgroup taxa.
25. *Simple neurochaetae [25]: absent [=0]; present [=1]*: Capillary neuropodial chaetae are present throughout *Pisione* and Sigalionidae*,* but absent in *Pisionidens*. Variable in outgroup taxa.
26. *Neurochaetal spines [26]: absent [=0]; present [=1]*: Stout, spike‐like chaetae are present in some species in the sigalionid genus *Pisione* (e.g., Govaere & De Wilde, 1993). Inapplicable for *Pisionidens*. Variable in outgroups.
27. *Unilateral fringed neurochaetae [27]: absent [=0]; present [=1]*: Series of setae, sometimes comb‐like (pectinate), along the blade and shaft. Inapplicable for *Pisionidens*. Variable in outgroup taxa.
28. *Compound falcigerous neurochaetae [28]: absent [=0]; present [=1]*: Compound chaetae with distally curved blades are exclusively found within Sigalionidae, as well as in some of outgroups. Blades and shafts may possess ornamentation and vary in size throughout the neuropodia. Present in *Pisione,* and scored as inapplicable for *Pisionidens*. Only present in outgroup taxa of Sigalionidae.
29. *Compound spinigerous neurochaetae [29]: absent [=0]; present [=1]*: Chaetal blades tapered distally. Usually present as a bifid tip with deep indentation between. Present in *Pisione* (e.g., Pettibone, 1970). Only present within outgroup taxa of Sigalionidae. Inapplicable for *Pisionidens*.
30. *Elytra [30]: absent [=0]; present [=1]*: Elytra are absent in *Pisione* and *Pisionidens*. Variable in outgroup taxa.
31. *Midventral pores [31]: absent [=0]; present [=1]*: Midventral pores are present in *Pisionidens*, are present only in males in some species, and are thought to be associated with reproduction and/or serve as an adhesive/glandular structure. Absent in *Pisione*. Absent in all outgroup taxa.
32. *Protruding notoacicula [32]: absent [=0]; present [=1]*: Internal aciculae occasionally are found with outermost tips protruding out from the notopodia. Variable in *Pisione* and Sigalionidae. Variable in outgroup taxa.
33. *Infra‐acicular simple chaetae [33]: absent [=0]; present [=1]*: In *Pisione*, the infra‐acicular chaetae is the third chaetae, counting from the dorsal‐most chaetae on the neuropodia (Yamanishi, 1998). Variable in *Pisione*. Absent in outgroups.
34. *Elongation of dorsal cirri on segment 3 [34]: absent [=0]; present [=1]*: Dorsal cirri of segment 3 may be elongated in comparison with that of neighboring cirri of other segments. Variable within the studied taxa of *Pisione*, present in all *Pisionidens* taxa. Absent in outgroup taxa.
35. *Prechaetal lobes divided [35]: absent [=0]; present [=1]*: In *Pisione,* the most distal part of the neuropodia may be divided into lobes or be whole. Variable in *Pisione*. Absent in outgroups.
36. *Long‐bladed compound chaetae [36]: absent [=0]; present [=1]*: Blades of one or more compound chaetae may present longer than other compound chaetae. Variable in *Pisione*. Variable in outgroups.
37. *Fusion of copulatory organ and parapodial lobe [37]: absent [=0]; partial [=1]*: In some *Pisione,* the copulatory organ proper merges with the parapodial stem, supported by aciculae, with few chaetae and no modification to the dorsal cirri. Absent in outgroups.
38. *Spiral structure of the copulatory organ [38]: absent [=0]; present [=1]*: Elongation of the copulatory structure causes a rotation to occur subterminally, with the distal end forming the cuticular penis. Variable in *Pisione*. Absent in outgroups.
39. *Inferior stem of the copulatory organ derived from bidigitate process [39]: absent [=0]; present [=1]*: A secondary stem that arises as the copulatory organ proper elongates. Projecting laterally, this inferior projection is connected by a thin membrane to the other copulatory stems. Variable in *Pisione*. Absent in outgroups.
40. *Bidigitate process of the copulatory organ homologous to inferior stem [40]: absent [=0]; present [=1]*: Derivation of character 41, where the outer digit or process of the copulatory organ attaches to the lateral side of the spiral structure, forming a bifurcated end. Variable in *Pisione*. Absent in outgroups.
41. *Sheath‐like arc of the copulatory organ [41]: absent [=0]; present [=1]*: A protrusion derived from the outer margin of the spiral structure, acting like a sheath, surrounding the penis. Variable in *Pisione*. Absent in outgroups.
42. *Cuticular plate of the copulatory organ [41]: absent [=0]; present [=1]*: Erected from the superior stem of the copulatory organ, covering the spiral region anteriorly. May be wrinkled along outer margin. Variable in *Pisione*. Absent in outgroups.
43. *Elongated ventral cirri of copulatory segments [43]: absent [=0]; present [=1]*: Ventral styles on copulatory segments are longer than those of non‐copulatory segments. Variable in *Pisione*. Absent in outgroups.
44. *Spinous papilla present on copulatory structures [44]: absent [=0]; present [=1]*: Spinous‐like papillae projecting on surfaces of the copulatory organ. Typically present in most distal regions; may form in circular masses. Variable in *Pisione*. Absent in outgroups.
